# Identification of Promising Candidate Genes and Immune Infiltration Patterns in Chronic Schistosomiasis-Associated Liver Injury by WGCNA and Machine Learning

**DOI:** 10.3390/pathogens15080806

**Published:** 2026-07-31

**Authors:** Yinlong Li, Qin Li, Suying Guo, Shizhu Li, Jing Xu

**Affiliations:** 1 Chinese Center for Disease Control and Prevention (Chinese Center for Tropical Diseases Research), National Key Laboratory of Intelligent Tracking and Forecasting for Infectious Diseases, National Institute of Parasitic Diseases, National Health Commission Key Laboratory of Parasite and Vector Biology, WHO Collaborating Centre for Tropical Diseases, National Center for International Research on Tropical Diseases, Shanghai 200025, China; liyl@nipd.chinacdc.cn (Y.L.); liqinchina@163.com (Q.L.); guosy@nipd.chinacdc.cn (S.G.); lisz@chinacdc.cn (S.L.); 2 School of Global Health, Chinese Center for Tropical Diseases Research, Shanghai Jiao Tong University School of Medicine, Shanghai 200025, China

**Keywords:** immune infiltration, chronic schistosomiasis, liver injury, differentially expressed genes (DEGs), GSEA, signaling pathway

## Abstract

**Background:** Dysregulated immune cells contribute to *Schistosoma japonicum*-induced liver injury. However, the underlying mechanisms remain poorly understood. This study aimed to identify feature genes and immune infiltration patterns linked to chronic schistosomiasis-associated liver injury. **Methods:** Differential gene expression analysis was conducted using dataset GSE61376 from the Gene Expression Omnibus (GEO) database. Functional enrichment of differentially expressed genes (DEGs) was performed via Gene Ontology (GO) and Kyoto Encyclopedia of Genes and Genomes (KEGG) analysis. Weighted Gene Co-expression Network Analysis (WGCNA) was applied to construct further characterization of molecular networks. LASSO regression and random forest algorithms were combined to screen hub genes, whose diagnostic efficacy was assessed by ROC analysis. CIBERSORT estimated immune cell infiltration, and GSEA explored pathways associated with hub genes. **Results:** A total of 412 DEGs were identified between chronic schistosomiasis and control groups. The green module from WGCNA exhibited significant correlation with chronic schistosomiasis (r = −0.85, *p* < 0.05). ANKMY2 and FCER1A were identified as hub genes with AUC values of 0.875 (95% CI, 0.500–1.000) and 0.792 (95% CI, 0.458–1.000). GSEA revealed associations with cytokine–receptor interaction and other signaling pathways. A total of 11 differentially distributed immune cell subsets were observed, and hub genes were correlated with multiple immune cell populations. **Conclusions:** ANKMY2 and FCER1A participate in liver injury of chronic schistosomiasis by regulating the hepatic immunopathological microenvironment. These two genes may serve as promising targets for immunotherapy against *S. japonicum*-induced liver injury.

## 1. Introduction

Liver injury triggers the progressive histopathological process initiated by dysregulated tissue repair programs in response to chronic hepatocyte injury, with major pathologically etiological drivers encompassing genetic predisposition, persistent viral infections (HBV/HCV), alcohol-induced oxidative stress, bile acid toxicity in cholestatic disorders, etc. [1,2]. The maladaptive response is characterized by dysregulated accumulation of extracellular matrix (ECM) components, including fibrillar collagens (predominantly type I and III collagen), basement membrane collagen (type IV), fibronectin, laminin, elastin and hyaluronic acid. The maladaptive wound-healing response is characterized by dysregulated, excessive accumulation of fibrillar collagens (predominantly type I and III collagen), basement membrane collagen (type IV), fibronectin, laminin, elastin and hyaluronic acid that disrupt the hepatic architecture [1,2]. This fibrotic progression represents a pivotal precursor to cirrhosis, culminating in life-threatening sequelae including hepatocellular carcinoma (HCC), hepatic decompensation, and portal hypertension [3,4]. The pathogenesis of liver fibrosis is complex, including chronic inflammatory cascades, hepatocyte apoptosis, fibroblast activation, and collagen deposition [5,6]. In recent years, research regarding liver fibrosis of diverse etiologies has grown rapidly. Among them, liver fibrosis caused by *S. japonicum* infection has attracted particular attention [7,8].

Schistosomiasis is one of the neglected tropical diseases caused by infection with trematode flukes belonging to the genus *Schistosoma*, dominating in 78 countries and territories of Asia, Africa, the Middle East and the Americas [9,10]. Among schistosomes parasitizing human beings, *S. japonicum* is a zoonotic parasite, causing intestinal and hepatosplenic schistosomiasis in China, the Philippines and small pockets of Indonesia. Immunopathological reactions triggered by eggs of *S. japonicum* trapped within the intestinal wall (predominantly colon mucosa and submucosa) and intrahepatic portal venous tissues drive intestinal inflammatory lesions. A large proportion of deposited eggs fails to migrate into the intestinal lumen and embolizes hepatic portal branches; soluble egg antigens secreted by trapped hepatic eggs induce persistent granulomatous inflammation, activate hepatic stellate cells, and trigger excessive ECM deposition, ultimately leading to progressive periportal liver fibrosis and distorted hepatic lobular architecture. The pathological basis of liver fibrosis due to chronic schistosomiasis is the immune response caused by the host from continuous stimulation of soluble egg antigen (SEA) secreted from schistosome eggs which are deposited in tissues of infected individuals [11,12]; subsequently, it leads to chronic inflammation, which is mainly characterized by egg granuloma and tissue damage, and ultimately causes liver fibrosis [9,13]. A Th2-type immune response is predominantly triggered by *S. japonicum* infection, with subsequent secretion of IL-4, IL-5, and IL-13, which activate liver fibrosis and stimulate collagen synthesis [14].

Recent studies have revealed the roles of various mechanisms and signaling pathways in liver fibrosis due to *S. japonica* [15]. During the infection process, the extensive infiltration of inflammatory cells such as macrophages, eosinophils, and T lymphocytes in the liver exacerbates the damage and fibrosis process of liver tissue [16,17,18,19]. However, the immune infiltration mechanisms underlying chronic schistosomiasis and the associated genes are not well explored and need further investigation. Furthermore, it is crucial to identify differential genes and explore the potential mechanisms linked to chronic schistosomiasis-related liver fibrosis.

In our study, a systematic approach with multiple bioinformatics methods integrated with an integrative machine learning framework was employed to investigate the interaction between chronic schistosomiasis and liver injury by delving into feature genes and immune cell infiltration. The research would provide new insights for precision immunotherapy in schistosomiasis-induced liver injury.

## 2. Material and Methods

### 2.1. Data Sources

**Figure 1:**
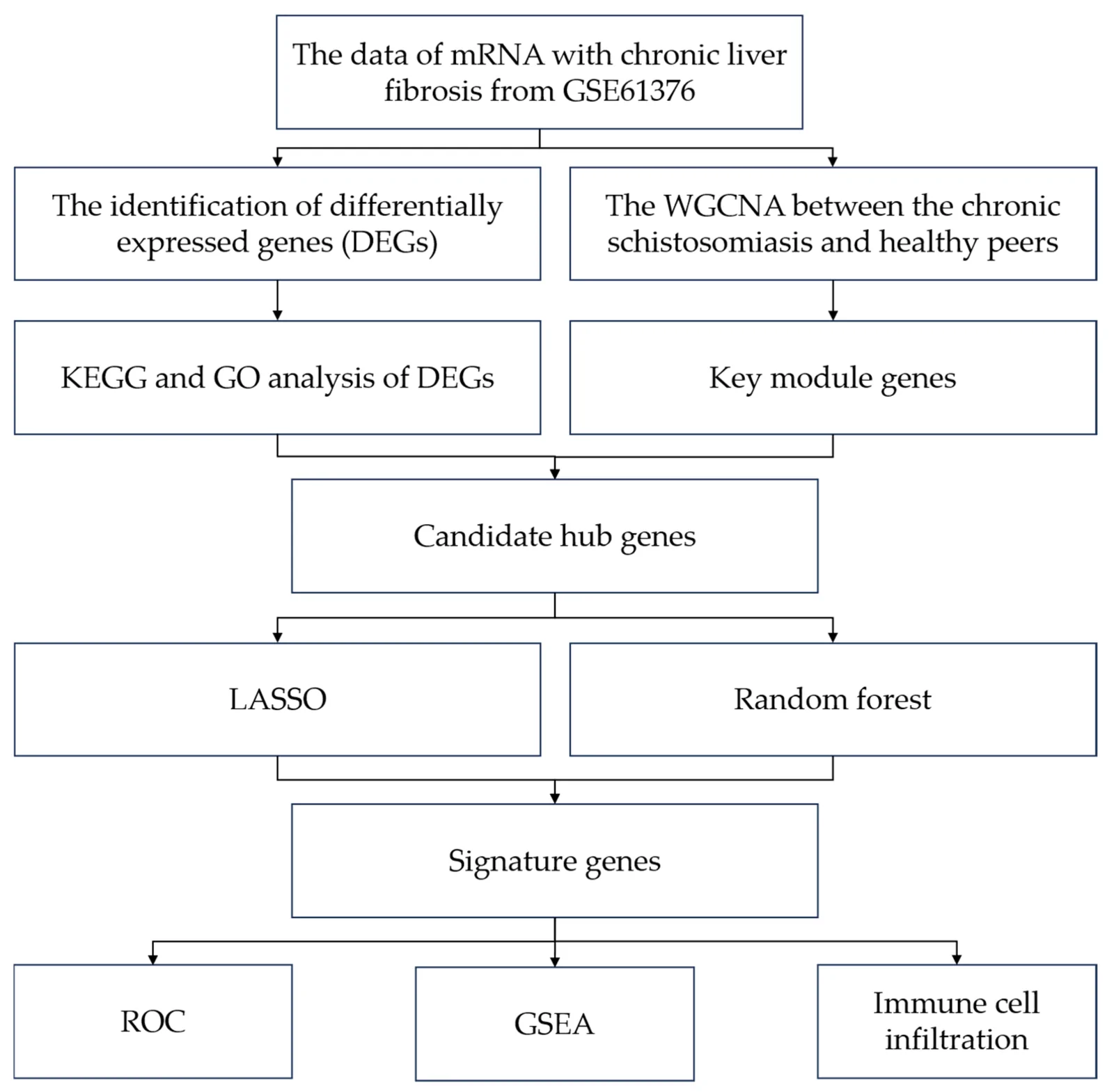
A flow diagram outlining the study design.

The gene expression profile of the human liver in individuals suffering from chronic infections of *S. japonicum* was investigated. A microarray analysis was conducted using cDNA synthesized from total RNA obtained from liver biopsy tissue collected in Hunan, China. Dataset of GSE61376 (Platform: GPL6947, Illumina) was downloaded from GEO www.ncbi.nlm.nih.gov/geo/ (accessed on 11 March 2024). The GSE61376 dataset comprises 17 individuals, including 4 healthy controls, 6 patients with chronic schistosomiasis and 7 individuals suffering schistosomiasis and hepatitis B simultaneously, but the last cohort was not included in this article because HBV co-infection causes confounding transcriptional noise that masks schistosomiasis-specific hepatic molecular changes. To explore the feature genes and immune cell infiltration in chronic schistosomiasis-associated liver injury, multiple bioinformatics methods integrated with machine learning algorithms were conducted on dataset of GSE61376. Figure 1 shows the flowchart illustrating this research.

### 2.2. DEGs Functional Annotation

Differentially expressed genes study was conducted between individuals diagnosed with schistosomiasis and those in control groups utilizing the limma package available in R software (version 4.1.2) [20]. Our analysis was guided by specific statistical criteria log fold change (FC) greater than 1 (adj *p*-value < 0.05). To visualize the DEGs, a volcano plot was created. Additionally, the top 100 genes exhibiting the highest levels of up-regulation, as well as the top 100 genes with the greatest down-regulation, were highlighted in a heatmap. The analysis of functional enrichment for the DEGs was conducted with the help of R packages, utilizing both GO and KEGG pathways as references [21].

### 2.3. Weighted Gene Co-Expression Network Analysis (WGCNA)

GSE61376 cohort was analyzed through the application of WGCNA using the criterion of scale-free topology [22]. The “pick Soft Threshold” function from the WGCNA package was used to determine the soft threshold power along with the related adjacencies. Following this, the adjacency matrix was transformed into a topological overlap matrix (TOM), from which dissimilarity was computed to conduct hierarchical clustering during gene analysis. To pinpoint the co-expressed gene modules, the dynamic tree cutting approach was employed, set with a minimum module size of 50. Additionally, the relationship between the gene modules and individuals suffering from schistosomiasis was evaluated by examining gene significance (GS) values alongside module membership (MM) values, which led to the identification of crucial modules.

### 2.4. Signature Genes

Candidate hub genes were identified by intersecting DEGs with key module genes. An integrated machine learning framework combining LASSO regression with the Random Forest (RF) algorithm was conducted to identify candidate hub genes, ensuring high precision selection. The input matrix was a standardized 14 (genes) × 10 (samples) matrix, with rows representing the 14 screened feature genes and columns representing individual samples (6 chronic schistosomiasis patients and 4 healthy controls), after excluding the 7 comorbid subjects to eliminate confounding interference. The raw gene expression matrix was imported and formatted with gene symbols as row names and expression values as numeric entries. Duplicate gene records were collapsed using the “avereps” function in the “limma package” by averaging their expression values to eliminate redundancy. Samples were divided into control and treatment groups according to predefined lists, and corresponding expression profiles were extracted and combined into a unified matrix. Each sample was annotated with its group label to ensure traceability, and the processed dataset was formatted to guarantee consistent structure and valid data types. Finally, the standardized, well-organized matrix was exported and used as input for subsequent Lasso regression and machine learning analyses.

LASSO regression and Random Forest (RF) models were constructed using R software (version 4.1.2), strictly following the principles of reproducibility and standardization. The models were built based on the glmnet, randomForest, and caret packages, with key parameters and implementation details as follows: First, a fixed random seed (set.seed(12345)) was set to ensure the reproducibility of all analytical processes. For the LASSO regression model, the standardized gene expression matrix was transposed to meet the input requirements, where genes served as independent variables and grouping labels (Control/Treatment) were used as binary dependent variables. The model adopted a binomial distribution, and the optimal penalty parameter λ (λ.min (log(λ) = −4.5) was determined via 10-fold cross-validation with deviance as the evaluation metric; genes with non-zero regression coefficients were selected as candidate features.

For the Random Forest model, the transposed standardized matrix was used as input, and the grouping information was converted into categorical variables. A 5-fold cross-validation was used to optimize the mtry parameter (candidate range: 1~10), and the initial model was trained with 500 decision trees (ntree = 500), which could be adjusted to 1000 decision trees to improve stability. Class weights proportional to group sizes were incorporated to alleviate sample imbalance. Feature selection was based on the Gini coefficient (Mean Decrease Gini), with a threshold of 2 to retain biologically meaningful genes; the top 10 key genes were selected for subsequent analysis. The Gini coefficient was used as the criterion for node splitting, and all model-related files were available via the designated platform to ensure traceability and reproducibility.

The genes that overlap between WGCNA and RF were considered characteristic genes related to chronic schistosomiasis-associated liver injury. To determine the diagnostic potential of candidate hub genes linked to liver injury, the area under the curve (AUC) for the receiver operating characteristic (ROC) curve was computed to evaluate the diagnostic effectiveness of these signature genes using the pROC package in R. AUC values exceeding 0.7 demonstrated strong discriminatory ability between fibrotic and non-fibrotic samples.

### 2.5. GSEA

In order to determine the relationship between signature genes and signaling pathways, we classified the schistosomiasis samples according to the median expression levels of hub genes and performed gene set enrichment analysis (GSEA) on the various subgroups (adj *p*-value < 0.05) [23].

### 2.6. Immune Cell Infiltration Analysis

The relative abundance of immune cell subtypes in liver tissues was quantified using the CIBERSORT algorithm, which employs a deconvolution approach based on gene expression signatures from the LM22 leukocyte reference matrix. Normalized expression data were filtered to retain only immune-related genes (ImmPort database) and subjected to 1000 permutations to ensure statistical robustness. Differential immune infiltration between fibrosis stages was assessed via Wilcoxon rank-sum tests, with false discovery rate (FDR) correction. Statistical analysis was performed using R software (version 4.1.2). Unless otherwise specified, a *p*-value < 0.05 was considered statistically significant, and all *p*-values were two-tailed.

## 3. Results

### 3.1. DEGs Between Schistosomiasis and Healthy Cohorts

**Figure 2:**
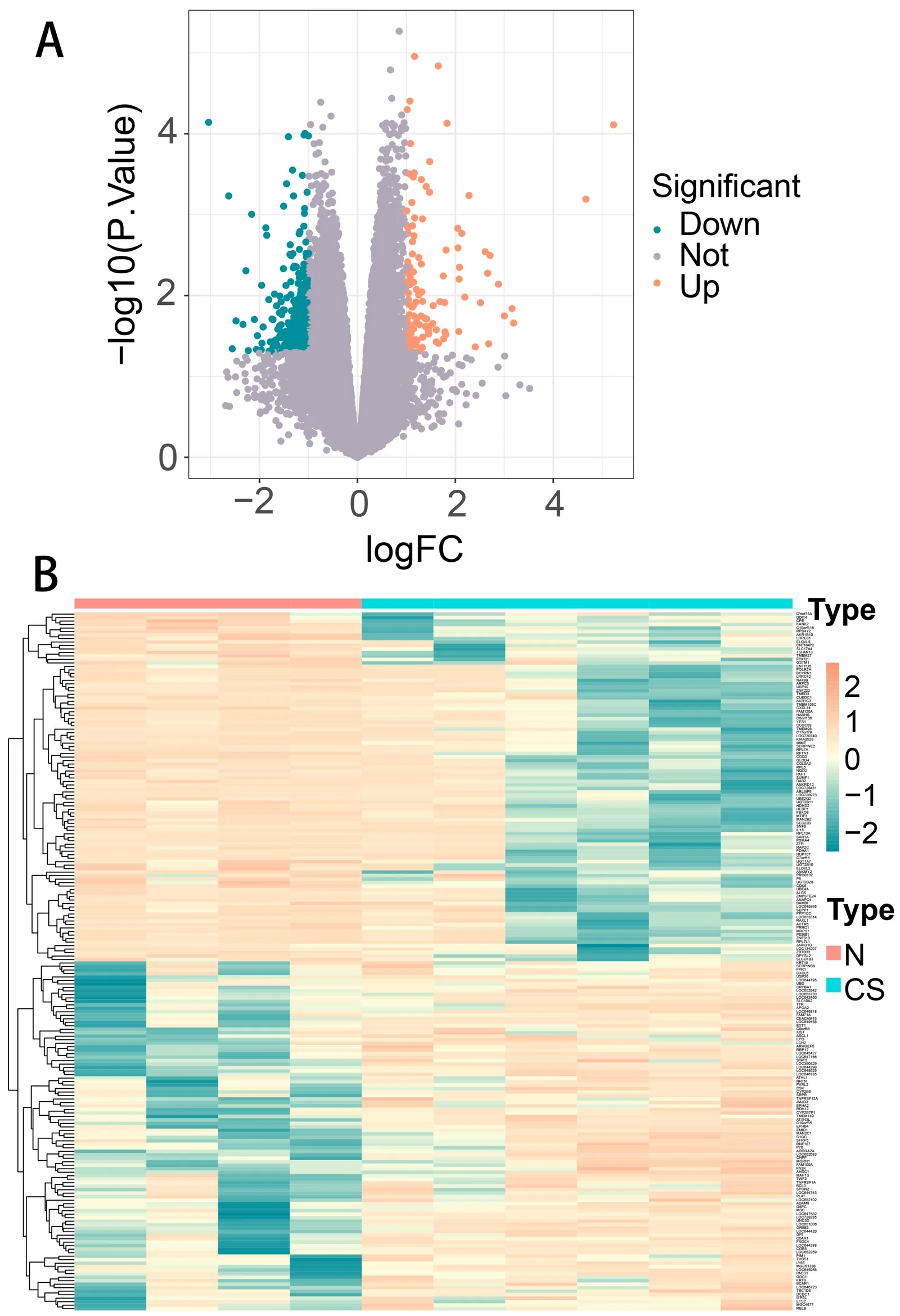
Identification of the differentially expressed genes (DEGs) in schistosomiasis. (**A**) The volcano plot illustrates the expression of DEGs in individuals with chronic schistosomiasis compared to normal controls. (**B**) 100 DEGs display (the top 50 up-regulated and 50 down-regulated).

All 412 DEGs (296 up-regulated and 116 down-regulated) were found in total by using “limma package” in R (Figure 2A). The heatmap illustrated the 100 DEGs display (the top 50 up-regulated and top 50 down-regulated) between chronic schistosomiasis and healthy groups (Figure 2B).

**Figure 3:**
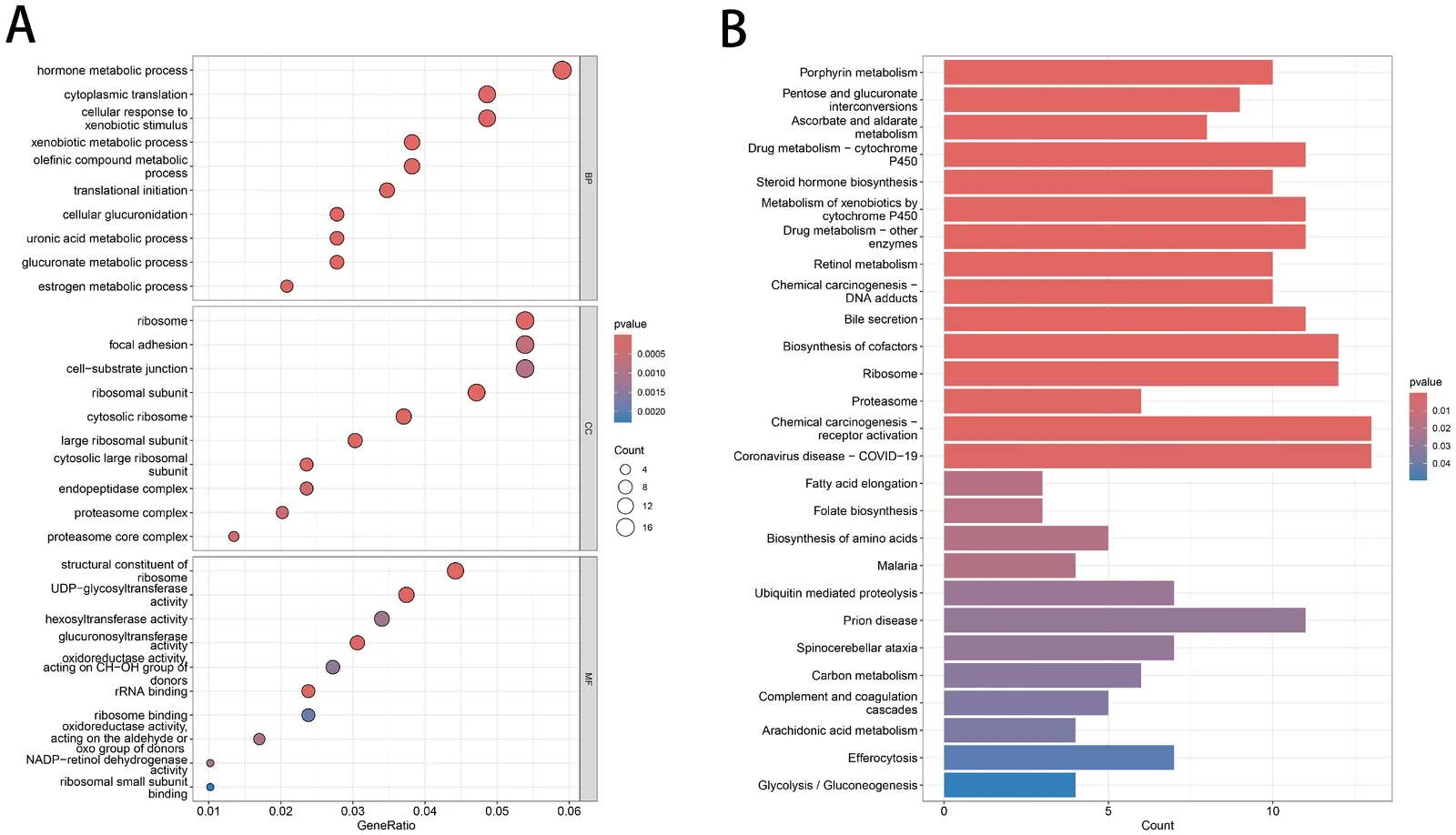
Functional enrichment analysis reveals key biological processes and pathways associated with chronic schistosomiasis-associated liver injury. (**A**) Top 10 results for BP, CC and MF. (**B**) Results of KEGG analysis.

The Gene Ontology (GO) study includes biological processes (BPs), cellular components (CCs), and molecular functions (MFs) (Figure 3A). The BP analysis identified significant enrichment in processes associated with the response to hormone metabolic processes, cytoplasmic translation, and cellular responses to xenobiotic stimuli. In the CC analysis, the top three enriched categories were ribosome, focal adhesion, and cell–substrate junction. Additionally, the MF analysis underscored the significance of structural constituents of the ribosome, UDP-glycosyltransferase activity, and hexosyltransferase activity. Figure 3B shows that the three most enriched pathways primarily associated with liver injury due to *S. japonicum* included porphyrin metabolism, pentose and glucuronate interconversions, and the ascorbate and aldarate metabolism signal pathway.

### 3.2. Module Construction and Hub Gene Screening

**Figure 4:**
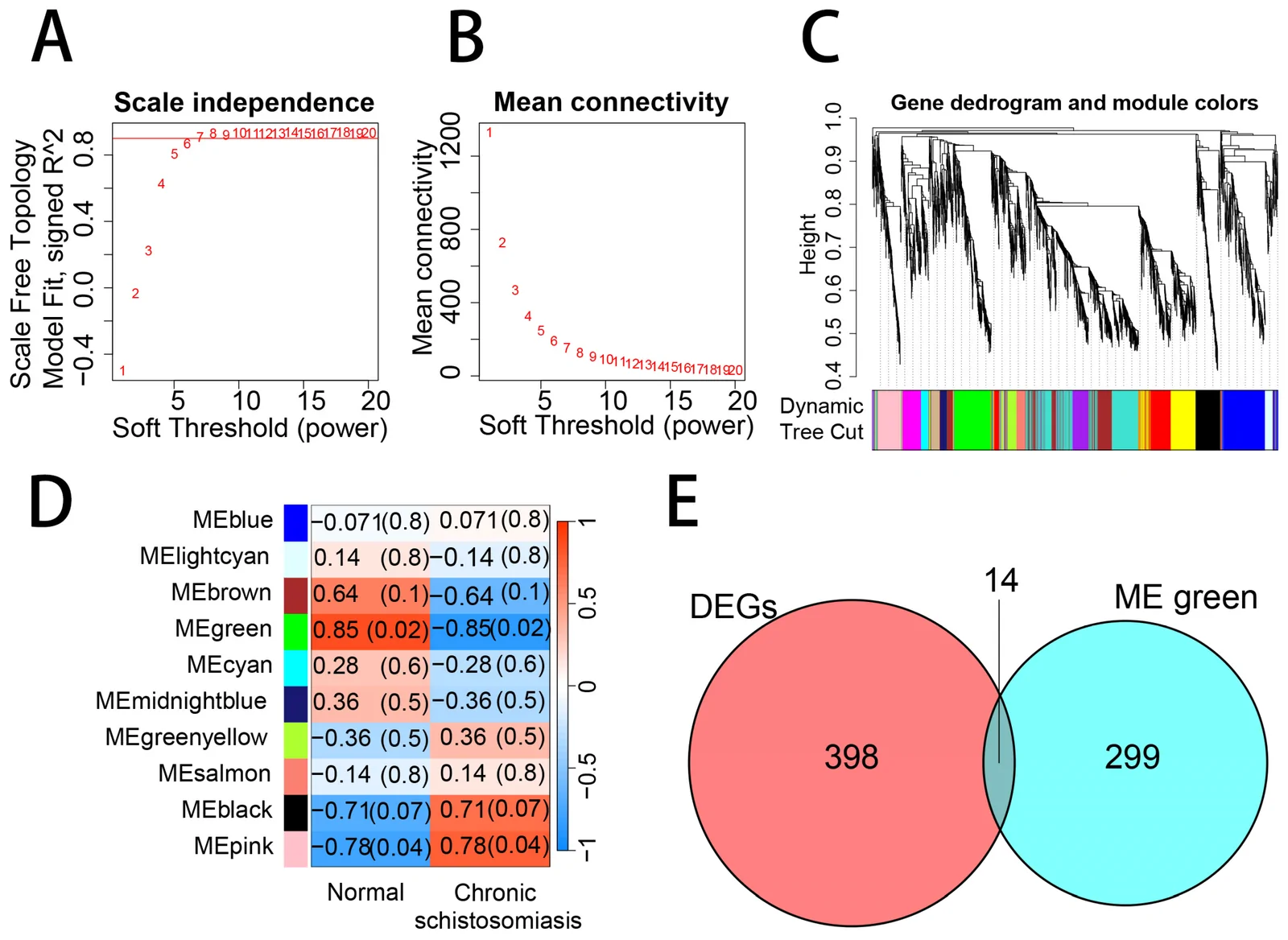
Analysis of GSE61376 and Hub Gene Screening. (**A**) The scale independence. (**B**) The mean connectivity. (**C**) Gene dendrogram and module colors. (**D**) Module–trait relationships. (**E**) The Venn plot of two parts (including DEGs and ME green module genes).

Chronic schistosomiasis and healthy subjects were analyzed using the WGCNA package in R software (version 4.1.2), resulting in the establishment of a scale-free co-expression network. The soft threshold power was set at 20. At this point, the scale-free index was 0.85 and the mean connectivity performance was superior (Figure 4A,B). Figure 4C depicted the cluster dendrogram findings. Relative data were grouped into eight modules (Figure 4D). The results showed that ME green module was significantly inversely correlated with chronic schistosomiasis (r = −0.85, *p* < 0.05). Additionally, the ME green module, which included 313 genes, was identified as a pivotal module related to individuals with schistosomiasis. All 14 genes, BEX4, EPO, SNX10, ARL4D, CANX, ANKMY2, GTF2IP1, FCER1A, JARID1B, UBE4A, PHCA, LOC203547, FAM45A and RPL11, were overlapped between two parts as shown in Figure 4E.

### 3.3. Signature Genes Selected by Integrative Machine Learning Approach

**Figure 5:**
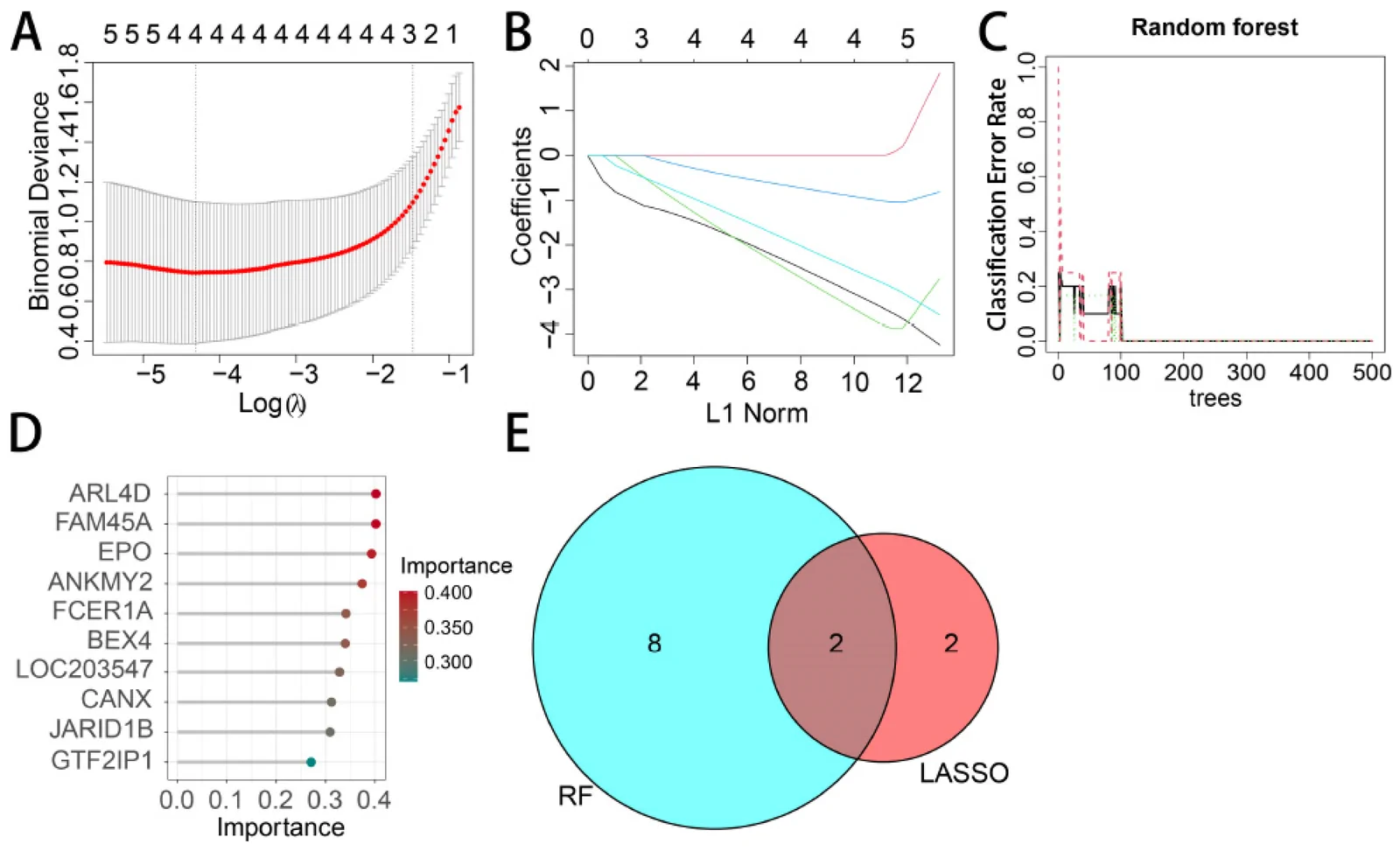
LASSO–Random Forest integrated analysis for hub gene identification. (**A**) LASSO regression binomial deviance profile (two vertical lines indicate the penalty parameters: the left dashed line represents lambda.min (log(lambda) = −4.5), which was selected as the optimal penalty parameter for key gene identification, and the right dashed line represents lambda.1se (log(lambda) = −2.5). A total of 4 non-zero coefficients were retained at lambda.min. (**B**) Coefficient profiles of LASSO regression. (**C**) Random Forest error convergence curve. (**D**) Gene importance ranking from Random Forest. (**E**) Overlap of hub genes identified by LASSO and Random Forest.

LASSO and RF were employed to identify characteristic genes. In this analysis, LASSO identified four promising candidate genes—ANKMY2, FCER1A, SNX10, and UBE4A—as key discriminative features (Figure 5A,B), likely through cross-validated coefficient optimization, and we selected λ_min_ (corresponding to log(λ) = −4.5) as the threshold for feature selection. Using this approach, ten promising candidate genes (ARL4D, FAM45A, EPO, ANKMY2, FCER1A, BEX4, LOC203547, CANX, JARID1B and GTF2IP1) were prioritized with the feature screening threshold > 2 (Figure 5C,D), indicating non-linear associations and potential interactions in the biological system.

The convergence of ANKMY2 and FCER1A across approaches confirms their biological and statistical significance. The consistency exhibited across different algorithms bolsters confidence that these factors can serve as promising candidate biomarkers, being robust to the assumptions made. ANKMY2 is a coding gene, and its encoded product is ankyrin repeat protein which is implicated in DNA repair and cancer progression. FCER1A is a protein-coding gene whose product serves as a high-affinity subunit of the IgE receptor that mediates allergic responses and immune activation. We observed their co-selection, which indicates the potential involvement of a mechanism related to our studied phenotype and several immune dysregulation diseases or cellular stress.

**Figure 6:**
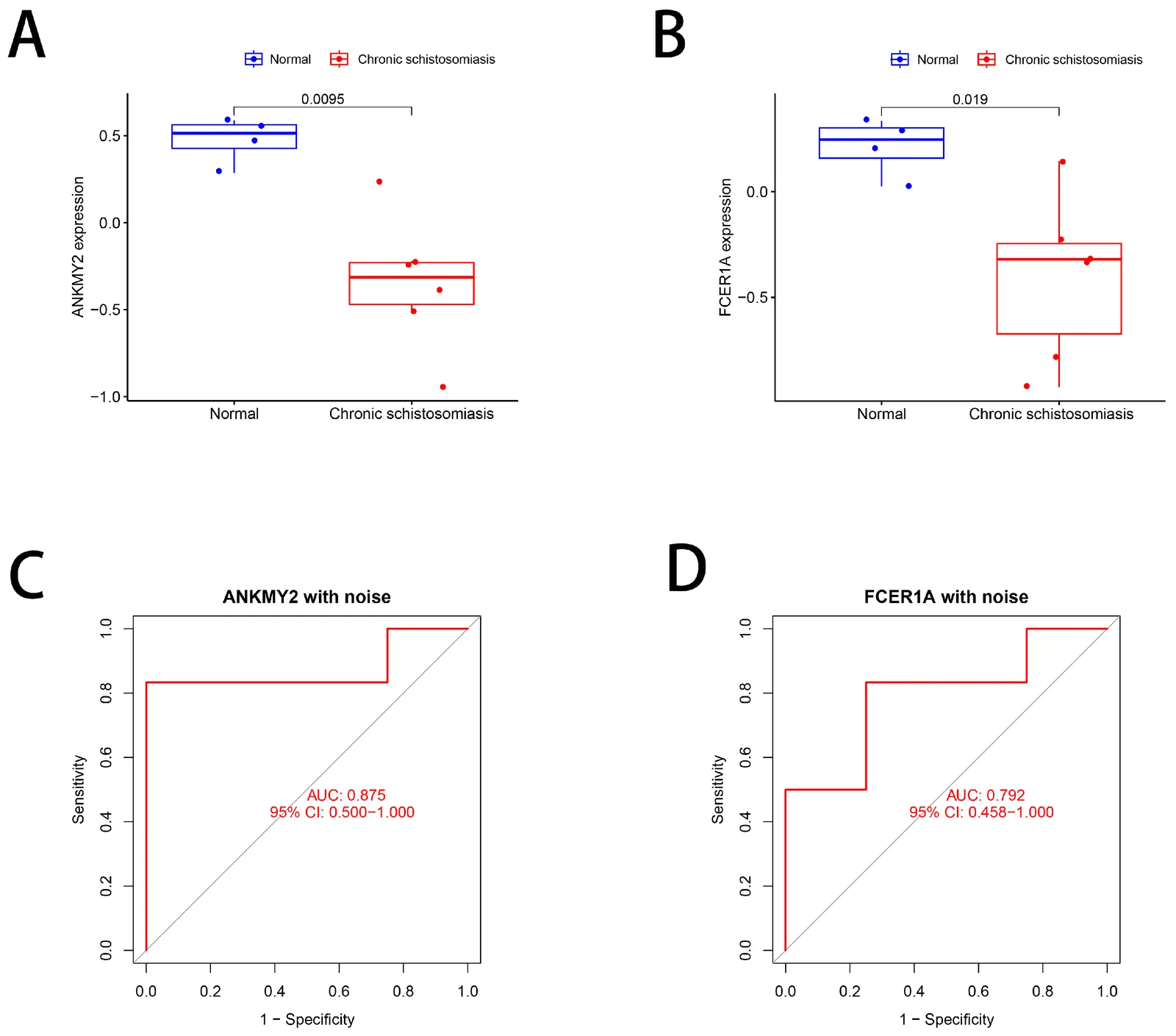
Expression differences and diagnostic efficacy of ANKMY2 and FCER1A in chronic schistosomiasis. (**A**) ANKMY2 expression in normal vs. chronic schistosomiasis groups. (**B**) FCER1A Expression in normal vs. chronic schistosomiasis groups. (**C**) ROC curve of ANKMY2 for chronic schistosomiasis diagnosis. (**D**) ROC curve of FCER1A for chronic schistosomiasis diagnosis.

The signature genes that were screened showed lower expression levels in chronic schistosomiasis than in normal control, indicating that the signature genes were contributing to the pathology of chronic schistosomiasis (Figure 6A,B). The AUC value of the receiver operating characteristic (ROC) for ANKMY2 and FCER1A was 0.875 and 0.792, respectively (Figure 6C,D). These findings suggested that these two signature genes exhibited significant efficacy in chronic schistosomiasis and may serve as potential candidates for diagnosis or therapeutics.

### 3.4. GSEA

**Figure 7:**
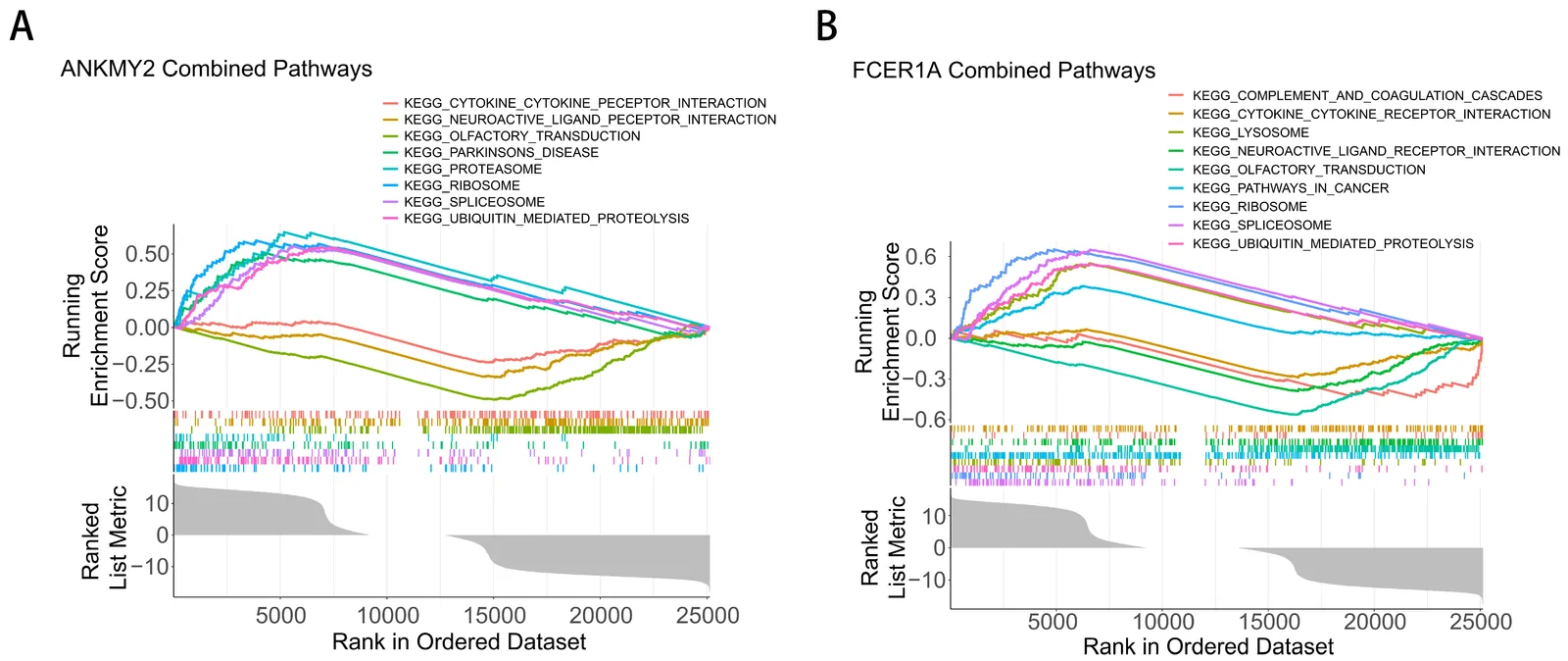
GSEA enrichment profiles of ANKMY2 and FCER1A-associated signaling pathways. (**A**) GSEA enrichment plot for ANKMY2-associated signaling pathways. (**B**) GSEA enrichment plot for FCER1A-associated signaling pathways.

Figure 7 shows that we performed GSEA enrichment analysis and further identified the top eight signaling pathways (*p*-values < 0.05). As presented in Figure 7, the ANKMY2 was significantly correlated with Parkinson’s disease, proteasome, ribosome, spliceosome, and ubiquitin-mediated proteolysis. The expression of FCER1A significantly correlated with lysosome, pathways in cancer, ribosome, spliceosome, and ubiquitin-mediated proteolysis. Collectively, the identified genes negatively correlate with ribosome, spliceosome, and ubiquitin-mediated proteolysis signaling pathways.

### 3.5. Immune Cell Infiltration

**Figure 8:**
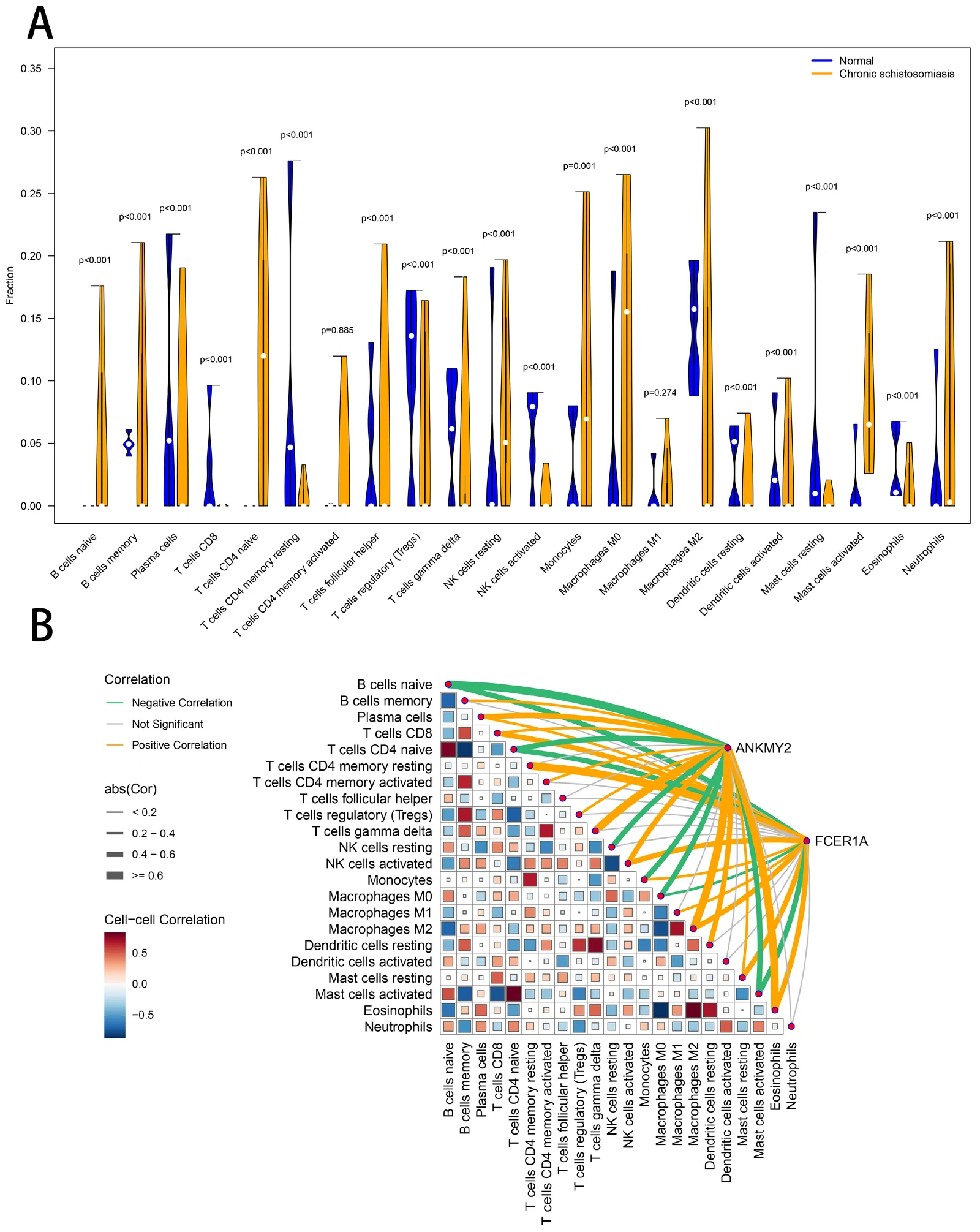
Immune cell infiltration analysis and gene–immune correlations in chronic schistosomiasis. (**A**) Immune cell infiltration profiles in normal vs. chronic schistosomiasis groups. (**B**) Correlation between immune cells and target genes (ANKMY2/FCER1A).

The immunological landscape was characterized via the infiltration profile of immune cells. As shown in Figure 8, compared with healthy controls, people with chronic schistosomiasis have higher naive B cells, M0 and M2 macrophages, etc. (with thick green lines) and lower CD8 T cells, T cells CD4 memory resting, and resting mast cells infiltration (with thick yellow lines) (Figure 8A). All identified genes were positively associated with CD8 T cells, plasma cells, M2 macrophages, activated NK cells, and eosinophils, while showing negative associations with naive CD4 T cells, naive B cells, and activated mast cells (Figure 8B).

## 4. Discussion

Being one of the most important organs in the human body, the liver plays a variety of functions primarily including metabolism, secretion, synthesis, biotransformation, and bile excretion. External and internal factors can cause liver dysfunctions, and even liver diseases. All liver diseases have complex pathological changes, which have been involved in the liver fibrosis response and have been researched for many years. Early liver fibrosis has been shown to be reversible. Therefore, timely intervention in the early stages of liver fibrosis to prevent further exacerbations of the disease is of great clinical significance.

Liver fibrosis induced by schistosomiasis is a complex pathological process [24,25], driven principally by the deposition of schistosome eggs in hepatic tissues. This process triggers a series of immune-mediated inflammatory responses by the release of soluble egg antigens [13,26]. Current scientific evidence demonstrates that these antigens potentiate systemic inflammatory cell infiltration [27,28], leading to the formation of egg granulomas and excessive deposition of extracellular matrix, ultimately manifesting as liver fibrosis, cirrhosis and in advanced stages, hepatocellular carcinoma. Notably, schistosomiasis infection promotes the recruitment and phenotypic activation of liver immune cells, including T cells, macrophages, and natural killer (NK) cells [29,30]. Immune cells produce pro-fibrogenic cytokines and chemokines to regulate inflammatory response and liver fibrosis progression [31,32,33]. Moreover, liver fibrosis is also associated with phenotypic and functional alterations of immune cells [34]. Recent studies have identified specific molecular mechanisms and signaling pathways underlying liver fibrosis induced by *S. japonicum* infection [10,35,36]. Additionally, an underlying feature of HBV/HCV infection is massive, persistent inflammatory cell infiltration—particularly macrophages, eosinophils and T lymphocytes—which aggravates hepatic injury and fibrotic progression [37,38]. Studying immune infiltration poses several difficulties owing to the complicated pathological changes in hepatic tissues, immune cell diversity, involvement of host factors, and differences existing between human and animal immune systems [39,40,41,42]. Identifying signature genes that affect immune cell infiltration is an important frontier of immunology and precision medicine in cancer, autoimmune diseases, and chronic inflammation. The proteins generated by these genes recruit immune cells and activate them in tissues, polarizing them and influencing their tissue functions. Germline polymorphisms in these genes may further explain interpatient variability in immune infiltration and possible immunomodulatory strategies. As the discipline evolves, systematically charting these genes will enhance predictive models, steer biomarker-driven trials, and unearth new ways to amplify the immune system for disease control.

The application of machine learning (ML) in genomics has facilitated the discovery of diagnostic and therapeutic biomarkers, particularly in complex multi-factorial diseases like liver fibrosis [43,44,45]. In this study, the use of a combination of two complementary ML algorithms, LASSO regression for feature selection and Random Forest for classification robustness, improved the accuracy of detecting signature genes from candidate hub genes. LASSO regression curbs over-fitting by imposing penalties on non-predictive variables, whereas ensemble methods like Random Forest enhance generalizability by aggregating repeated decision trees [46,47]. By employing these two strategies, we can prioritize genes that have high predictive power through rigorous data analysis. GSEA results of the screened candidate genes revealed significant enrichment in ribosome, spliceosome and ubiquitin-mediated proteolysis pathways. These pathways maintain intracellular protein homeostasis in hepatocytes [48]. Interference with spliceosomal RNA processing or ribosomal biogenesis, for example, may disrupt cellular repair functions, while faulty ubiquitin-mediated degradation may lead to the accumulation of misfolded proteins and trigger endoplasmic reticulum stress, a recognized fibrogenic driver [49,50]. Focusing on these eight core pathways enables our study to capture molecular processes linking metabolic dysfunction and immune activation, two characteristic pathological features of schistosomiasis-associated hepatic injury. The study of metabolic pathways is an exciting field of computational biology and has received much attention in recent times [51]. The identification of ANKMY2 and FCER1A as signature genes provides new insights into chronic schistosomiasis-related liver injury’s molecular mechanisms.

ANKMY2 is an ankyrin-repeat protein that has been shown to have an effect on both nuclear signaling and protein–protein interactions. Moreover, it may impact ribosomal assembly and spliceosomal activity [52]. Disruption of the mRNA splicing fidelity or the ribosome biogenesis may lead to the incorrect synthesis of proteins by HSCs, which is a crucial event for the activation of HSCs towards a collagen-secreting, pro-fibrotic phenotype [53].

On the other hand, there is evidence to suggest that FCER1A, an IgE receptor with high affinity, which encodes its alpha subunit, could relate to immune dysregulation and fibrotic progression [54]. FCER1A is involved in mast cell activation and IgE-mediated hypersensitivity responses and may contribute to the chronic inflammation observed in schistosomiasis, resulting from Th2-polarized immunity driven by Schistosoma eggs’ persistent antigenic stimulation [17,55]. The abundant pro-inflammatory mediators released in this process not only aggravate tissue damage, but also cooperate with dysregulated ubiquitin–proteasome signaling to boost extracellular matrix accumulation [56]. The pathway of proteolysis mediated by ubiquitin may regulate the turnover of fibrogenic regulators (e.g., TGF-β or TIMP proteins, or both), resulting in feedforward fibrosis [57]. Collectively, ANKMY2 and FCER1A, two key genes regulating immune and inflammatory responses, illustrate the vital roles of post-translational modification and protein function in disease progression. Furthermore, these two genes serve as promising candidate biomarkers for early diagnosis and evaluation of therapeutic response in schistosomiasis.

Subsequent ROC curve analysis showed that ANKMY2 and FCER1A could significantly distinguish chronic schistosomiasis patients from healthy individuals. The genes ANKMY2 and FCER1A could be ideal targets for regulating immune cell infiltration. Past studies indicated that ANKMY2 influences Shh signaling [58]. Nevertheless, no prior research has explored the diagnostic value and biological function of ANKMY2 and FCER1A in schistosomiasis-associated liver injury.

Exploration of core functional genes related to chronic schistosomiasis deepens our understanding of disease diagnosis, particularly for early fibrotic lesions. Moreover, the correlation between signature genes and tissue immune cells reflects the immune infiltration landscape of schistosomiasis-induced liver injury. Hepatic lesions caused by *S. japonicum* infection are characterized by prominent immune cell infiltration; clarifying this regulatory network is essential to elucidate disease pathogenesis and develop innovative therapeutic strategies. Our study provides new evidence for potential diagnostic and therapeutic targets of chronic schistosomiasis from the perspective of immune infiltration and related gene regulatory networks.

This study has several unavoidable limitations. First, all transcriptomic data were retrieved from public open-access databases with a relatively small sample size, which may introduce analytical bias. Therefore, the gene panel identified in our research should only be regarded as exploratory candidate biomarkers, and their reliability requires further validation in larger independent clinical cohorts before drawing definitive conclusions. Second, all findings of the present study are derived solely from bioinformatic prediction without in vitro or in vivo experimental verification. Subsequent functional experiments are required to validate our conclusions. Future research will construct gene knockout and overexpression mouse models, establish S. japonicum infection models, and further explore the regulatory relationships between these core genes, hepatic injury, immune cell infiltration and T cell activation. Second, the original public dataset lacks complete clinical metadata. Although we excluded patients co-infected with HBV/HCV to eliminate major viral confounding factors, records regarding alcohol consumption history, pesticide environmental exposure, and infection status of other viruses (e.g., HPV) were unavailable. These unmeasured risk factors capable of inducing liver injury could not be excluded, which may affect data interpretation.

## 5. Conclusions

Based on an integrated analysis by multiple bioinformatics methods and machine learning algorithms on an open-access dataset, this study identified two key genes, ANKMY2 and FCER1A, that exhibited significant relevance in the context of liver injury associated with chronic schistosomiasis. These findings suggest these genes may serve as potential candidates for novel precision immunotherapy targets in schistosomiasis-induced liver injury.

## Data Availability

Dataset of GSE61376 (Platform: GPL6947, Illumina) was downloaded from GEO (www.ncbi.nlm.nih.gov/geo/ accessed on 11 March 2024).

## Abbreviations

The following abbreviations are used in this manuscript:

DEGs	Differentially Expressed Genes
GEO	Gene Expression Omnibus
GO	Gene Ontology
KEGG	Kyoto Encyclopedia of Genes and Genomes
WGCNA	Weighted Gene Co-expression Network Analysis
RF	Random Forest
LASSO	Least Absolute Shrinkage and Selection Operator
HBV	Hepatitis B Virus
HCV	Hepatitis C Virus
ECM	Extracellular Matrix
HCC	Hepatocellular Carcinoma
FC	Fold Change
BP	Biological Process
CC	Cellular Component
MF	Molecular Function
TOM	Topological Overlap Matrix
GS	Gene Significance
MM	Module Membership
ML	Machine Learning
